# Cytoreductive surgery (CRS) and hyperthermic intraperitoneal chemotherapy (HIPEC) in small bowel adenocarcinoma with peritoneal metastasis: a systematic review

**DOI:** 10.1515/pp-2022-0121

**Published:** 2022-11-18

**Authors:** Vicky Chen, Morgan Jones, Lauren Cohen, Wilson Yang, Jasman Bedi, Helen M. Mohan, Sameer S. Apte, José Tomas Larach, Michael Flood, Alexander Heriot, Joseph Kong, Satish Warrier

**Affiliations:** Department of Surgical Oncology, Peter MacCallum Cancer Centre, Melbourne, VIC, Australia; Department of Colorectal Surgery, Alfred Health, Prahran, VIC, Australia

**Keywords:** cytoreductive surgery (CRS), hyperthermic intraperitoneal chemotherapy (HIPEC), peritoneal carcinomatosis, small bowel adenocarcinoma, small bowel cancer

## Abstract

**Objectives:**

Small bowel adenocarcinoma (SBA) with peritoneal metastasis (PM) is rare and despite treatment with systemic chemotherapy, the prognosis is poor. However, there is emerging evidence that cytoreductive surgery (CRS) with hyperthermic intraperitoneal chemotherapy (HIPEC) may offer a survival benefit over systemic therapy alone. This systematic review will assess the effectiveness of CRS–HIPEC for SBA–PM.

**Content:**

Three databases were searched from inception to 11/10/21. Clinical outcomes were extracted and analysed.

**Summary:**

A total of 164 cases of SBA–PM undergoing CRS–HIPEC were identified in 12 studies. The majority of patients had neoadjuvant chemotherapy (87/164, 53%) and complete cytoreduction (143/164, 87%) prior to HIPEC. The median overall survival was 9–32 months and 5-year survival ranged from 25 to 40%. Clavien–Dindo grade III/IV morbidity ranged between 19.1 and 50%, while overall mortality was low with only 3 treatment-related deaths.

**Outlook:**

CRS–HIPEC has the potential to improve the overall survival in a highly selected group of SBA–PM patients, with 5-year survival rates comparable to those reported in colorectal peritoneal metastases. However, the expected survival benefits need to be balanced against the intrinsic risk of morbidity and mortality associated with the procedure. Further multicentre studies are required to assess the safety and feasibility of CRS–HIPEC in SBA–PM to guide best practice management for this rare disease.

## Introduction

Neoplasms originating from the small intestine are rare, representing less than 5% of all gastrointestinal (GI) tract malignancies and less than 1% of all cancers [1]. Of the histological types, small bowel adenocarcinoma (SBA) and neuroendocrine tumours account for approximately 40% each, while gastrointestinal stromal tumours, sarcomas and lymphomas comprise the remaining 20% [2]. Due to initial non-specific symptoms and difficulty in assessing the entire length of small bowel with traditional endoscopy, patients with SBA are typically diagnosed at an advanced stage [3]. Approximately a third present with metastases, most commonly in the liver, followed by the peritoneal cavity and extra-regional lymph nodes [4], [5], [6]. Prognosis is usually poor with the combined 5-year overall survival (OS) rate of metastatic SBA being less than 20% [7].

Metastatic SBA with peritoneal metastasis (PM) is commonly treated with traditional fluoropyrimidine (5-FU) or 5-FU plus oxaliplatin based systemic palliative chemotherapy, but the overall life expectancy remains poor [8]. Comparative studies between systemic therapy vs. no treatment show an objective response rate between 6 and 50% and median OS of 9–16 months [9], [10], [11], [12]. In the absence of randomised trials, there remains a lack of consensus on the preferred first line systemic chemotherapy regimen for SBA–PM [13].

An alternative treatment for SBA–PM is cytoreductive surgery (CRS) combined with hyperthermic intraperitoneal chemotherapy (HIPEC). CRS involves the surgical excision of all intra-abdominal macroscopic disease. HIPEC provides the cytotoxic effect of hyperthermia as well as regional dose intensification through chemoperfusion to treat the remaining microscopic disease within the abdominal cavity. Agents are generally left intraperitoneally for 30–90 min to allow mixing and contact with tumour cells. CRS–HIPEC has been shown to improve survival in selected patients with PM from colorectal [14] and ovarian cancer [15, 16]. It has also been suggested that CRS–HIPEC could be a superior treatment to systemic therapy as a single treatment in appropriately selected patients with SBA–PM [13]. The purpose of this study is to systematically review the existing literature on the effectiveness of CRS–HIPEC in the treatment of patients diagnosed with SBA–PM.

## Methods

**Figure 1:**
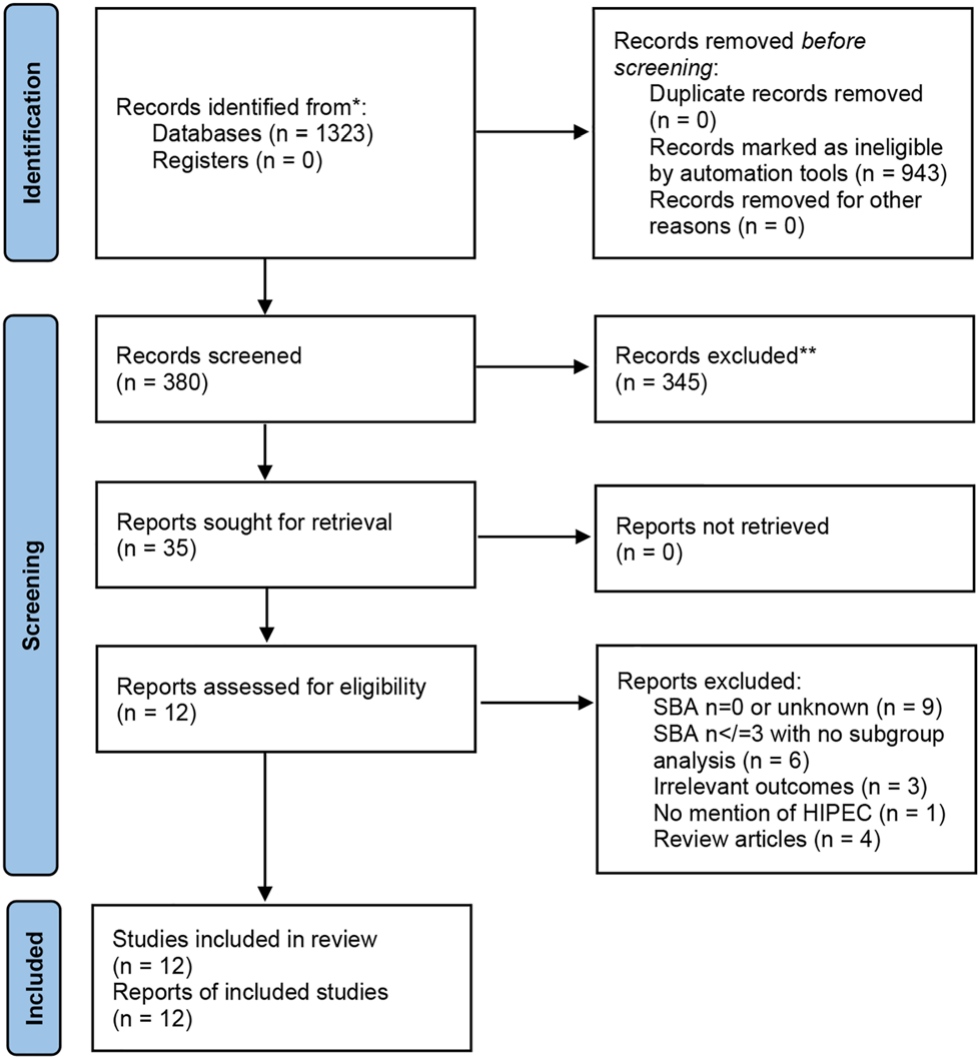
PRISMA flowchart showing search results for the systematic review.

A systematic review was performed with reference to the PRISMA statement (Figure 1 – PRISMA flow diagram). The research question and inclusion and exclusion criteria were developed *a priori* and registered with PROSPERO (Reference ID – 277721) to commencement.

### Inclusion and exclusion criteria

All published studies to date that reported on patients who received CRS–HIPEC for SBA–PM were included in this review. Studies were excluded if they were case reports with 3 patients or less, if the small bowel adenocarcinoma cohort could not be separated from other pathologies, or if they were review articles or conference abstracts.

### Outcomes

The primary outcomes were firstly, progression-free survival (PFS), which is the length of time during and after the treatment of a disease that a patient lives with the disease but it does not get worse, and secondly, overall survival (OS) following CRS–HIPEC. This was calculated from the time of the index CRS–HIPEC procedure to death. Secondary outcomes included: overall morbidity, type of morbidity, treatment-related deaths, mean length of hospital stay and quality of life score.

### Search methods

A systematic literature search was conducted using PubMED/Medline, EMBASE and Web of Science using a predefined search strategy (Appendix 1) on 11 October 2021.

### Data extraction and management

Two reviewers (VC and LC) independently screened the studies derived from the search and selected the pertinent articles to retrieve according to title and abstract. Full text versions of the retrieved articles were assessed to select the relevant articles which met all specified inclusion criteria. Discrepancies were resolved by discussion with a 3rd and 4th reviewer (HM and SA). If available, the following outcome parameters were extracted and recorded in a spreadsheet: study characteristics, patient demographics (age, gender), details of intervention (HIPEC technique, extent of CRS), survival outcomes (progression-free and overall survival), mortality and morbidity data (length of hospital stay, treatment-related deaths, Clavien–Dindo III/IV morbidity, treatment-related deaths) and quality of life.

### Statistical analysis

**Figure 2:**
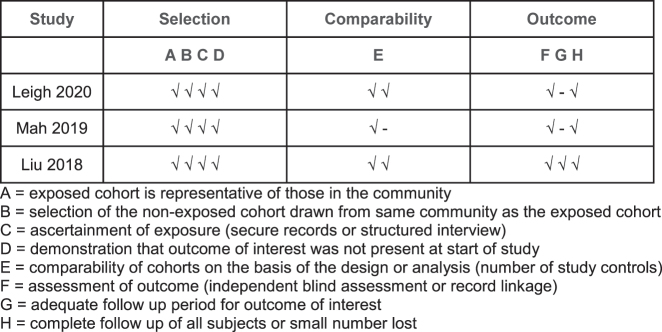
Newcastle–Ottawa quality assessment for the included studies.

Descriptive statistics (counts, means and medians) were used to report study, patient and treatment data. Data for patients that underwent CRS–HIPEC were pooled to analyse the efficacy of this intervention compared to systemic chemotherapy alone and presented in a descriptive format. A meta-analysis was unable to be performed as there was no comparator. The quality of the included studies was assessed using the Newcastle-Ottawa Scale (Figure 2).

## Results

### Results of the search

Our search criteria identified 380 independent articles (Figure 1). After screening by title and abstract, 35 were retrieved for full-text review. Assessment of the 35 articles by 2 independent reviewers identified a total of 12 studies which met the inclusion criteria. These included:

– 7 prospective observational studies: 4 single-centre [17], [18], [19], [20] and 1 multicentre [21]
– 5 retrospective studies: 4 single-centre [22], [23], [24], [25] and 3 multicentre [26], [27], [28]

However, closer examination of the 12 studies identified overlap in the reporting of a number of patients with SBA–PM undergoing CRS–HIPEC. Six patients in Jacks et al.’s 2005 paper are re-reported in Sun et al.’s 2013 paper, while Liu et al.’s 2018 publication includes all the patients reported across the preceding 9 published studies. If patient populations or studies were repeated within different papers, they were only included once to ensure no overlap.

### Characteristics and quality of included studies

A total of 309 patients were presented in the 12 studies and numbers of patients in each study ranged between 4 and 152 (Table 1). After accounting for the overlapping patients between single centre and multicentre studies, 164 cases of SBA–PM undergoing CRS–HIPEC were identified. These were reported across 3 studies [22, 23, 28]. These 3 non-randomized studies scored 7 or more on the Newcastle-Ottawa scale and were deemed good quality studies (Figure 2). The median age ranged from 54 to 58 with a preponderance of male (54–88%) patients and peritoneal cancer index (PCI) ranging from 10 to 21.5.

**Table 1:** Baseline characteristics in included studies without accounting for overlap.

Study	Country	Study design	No of patients	Median age, years	Gender (% male)	Diagnosis	Median PCI	Perioperative systemic chemotherapy, %	Median length of follow-up, months
Leigh 2020	USA	Retrospective, single-centre	8	58	88	SBA + PM	11 (median)	Neoadjuvant 63%, adjuvant 63%	9
Mah 2019	Canada	Retrospective, single-centre	4	NS	NS	SBM + PM	21.5 (mean)	NS	NS
Liu 2018	Multi-national	Retrospective, multicentre (21 centres)	152	54	54	SBA + PM	10 (median), 12 (mean)	Neoadjuvant 54%, adjuvant 53%	20
Legue 2017	Netherlands	Retrospective, multicenter	15	53	NS	SBA + PM	NS	NS	NS
Saxena 2017	Australia	Retrospective, single-centre	16	51.6	63	SBA + PM	11 (mean)	Neoadjuvant 25%, adjuvant 94%	21
Liu 2016	Japan	Prospective, single-centre	31	56	48	SBM + PM	NS	Neoadjuvant 90%	36
Van Oudheusden 2015	Netherlands	Prospective, multicenter	16	60.9	19	SBA + PM	NS	Adjuvant 56%	17
Sun 2013	USA	Prospective, single-centre	17	52.2	41	SBA + PM	NS	Neoadjuvant 76%, adjuvant 29%	NS
Elias 2010	France	Retrospective, mutlicentre	31	NS	61	SBM + PM	11 (median), 11 (mean)	Adjuvant 55%	45
Chua 2009	Australia	Prospective, single-centre	7	47	71	SBA + PM	12	Neoadjuvant 14%	17
Jacks 2005	USA	Retrospective, single-centre	6	50	17	SBA + PM	NS	Neoadjuvant 67%, adjuvant 50%	NS
Marchettini 2002	USA	Prospective, single-centre	6	46.5	67	SBA + PM	NS	NS	NS

NS, not specified; SBM, small bowel malignancy; SBA, small bowel adenocarcinoma; PM, peritoneal metastasis; PCI, peritoneal cancer index.

### Selection of patients

Leigh et al.’s retrospective cohort study of 8 patients from a single institution selected patients with confirmed adenocarcinoma histology and excluded patients with radiographic evidence of extra-peritoneal metastases. Mah et al. also presented a single-centre retrospective cohort study but did not state whether all 4 included patients with small bowel malignancy had adenocarcinoma histology. This study excluded patients who had previously undergone HIPEC. The largest study came from Liu et al. who drew on a multi-institutional data registry on SBA–PM treated with CRS–HIPEC. The 152 patients in this study had confirmed adenocarcinoma histology. Mah et al. and Liu et al. calculated PCI during the CRS–HIPEC procedure while Leigh et al.’s patients had their PCI calculated prior to the procedure.

### Perioperative systemic chemotherapy

With regards to systemic chemotherapy, Leigh et al.’s study reported that 63% of patients received neoadjuvant chemotherapy in the form of FOLFIRI, and the same number underwent adjuvant therapy. Liu et al. showed 53.9 and 53.3% of patients received neoadjuvant and adjuvant therapy respectively comprising of FOLFOX, FOLFIRI, XELOX or TS-1. One study (Mah) did not include any data on systemic chemotherapy. Overall, a similar proportion of patients received neoadjuvant (87/164, 53%) and adjuvant (86/164, 52.4%) chemotherapy.

### Surgical outcomes and HIPEC regimen

The majority of patients (143/164, 87%) had complete cytoreduction (CC-0/1) prior to HIPEC (Table 2). Most centres adopted an open HIPEC technique (13/23, 57%), most commonly using mitomycin C (81/164, 49.4%) or oxaliplatin regimens (76/164, 46.3%) within the abdomen. The HIPEC duration ranged from 30 to 120 min with a median length of 60 min. Intra-abdominal temperatures were quoted as between 40 °C and 43 °C. Early post-operative intraperitoneal chemotherapy (EPIC) was added to supplement microscopic tumour treatment for 12 patients in the studies quoted (7.3%).

**Table 2:** Extent of CRS and HIPEC regimen in included studies.

Study	Percentage of CC-0/1^a^ (%)	HIPEC technique	Duration of HIPEC, min	Intra-abdominal temperature, °C	HIPEC regimen (numbers)	EPIC, %
Leigh 2020	62.5	Closed	90	42	Mitomycin C [8]	NS
Mah 219	100	NS	60	40–42	Oxaliplatin [4]	NS
Liu 2018	88.2	Open (13 centres), closed (8 centres)	30–120	41–43	Mitomycin C (73), oxaliplatin (72), other^b^ [7]	12 (7.8%)

^a^CC, completeness of cytoreduction; CC-0, no peritoneal disease seen; CC-1, persisting disease <2.5 mm. ^b^Other=1 doxorubicin, 1 docetaxel + cisplatin, 1 doxorubicin + cisplatin, 1 doxotaxel.

### Survival

Of the 164 patients, the median OS was 9–32 months. Five-year OS rates ranged from 25 to 38%. Further details on survival are provided in Table 3.

**Table 3:** Survival following CRS + HIPEC for SBA–PM.

Study	Median PFS, months	Median overall survival, months	1-year OS, %	3-year OS, %	5-year OS, %
Leigh 2020	7	9	38	38	38
Mah 2019	NS	25.4	75	25	25
Liu 2018	14	32	83	46	31

### Mortality and morbidity outcomes

Only 3 treatment-related deaths were reported from a total of 164 patients (Table 4). Two patients died from multiorgan failure and disseminated intravascular coagulation within 60 days of undergoing CRS–HIPEC, and one died of respiratory failure 84 days after surgery [28]. Between 19.1 and 50% of patients experienced a Clavien–Dindo grade III/IV morbidity, the most common of which was intraperitoneal abscess, followed by septicemia and intestinal fistula.

**Table 4:** Mortality and morbidity following CRS + HIPEC for SBA–PM.

Study	Median length of hospital stay, days	Treatment-related deaths	Overall morbidity, %	Clavien–dindo morbidity III/IV, %
Leigh 2020	14	0	NS	4/8 (50)
Mah 2019	NS	0	NS	2/7 (34.2)
Liu 2018	16	3^a^	39.5	29/152 (19.1)

^a^Cause of death – 1 multiorgan failure at D35, 1 disseminated intravascular coagulation at D49, 1 pulmonary failure at D84

### Quality of life

Only one study from the initial literature search documented quality of life after CRS and HIPEC, however, only 2 of the participants in this report had a small bowel malignancy [29]. This study was excluded from the review due to a lack of stratification of results by different tumour types, thus precluding analysis of the small bowel cohort separately.

## Discussion

In this systematic review, the reported cases of SBA-PM are small, signifying the rarity of the condition. The collated studies show that CRS–HIPEC may offer a survival benefit in a highly selected group of patients with SBA–PM. Longer survival was associated with well-differentiated tumours [28], absence of positive lymph nodes [26, 28], lower PCI scores of less than or equal to 15 [23, 25, 28], complete cytoreduction [21, 23, 28], age less than 70 [27] and undergoing CRS–HIPEC within 6 months of diagnosis [19, 28]. These studies suggest that patients with less aggressive disease and lower peritoneal burden are more amenable to complete macroscopic resection and would be better suited to undergo CRS–HIPEC. Younger patients with fewer pre-existing comorbidities may be able to undertake CRS–HIPEC sooner after diagnosis, which may be associated with better survival outcomes. The median age in the largest study assessed was 54 with a median PCI of 10 [28]. Whilst no consensus exists with regards to patient selection nor suitability, it has been demonstrated that morbidity and mortality of CRS–HIPEC increases with advanced age [30, 31].

SBA is a rare entity with a lifetime risk of developing SBA 2–5 times less than the risk of developing CRC in the US [32]. Whilst the incidence of SBA is less than CRC, the prognosis is worse, especially for primary pathology in the duodenum. Five-year OS for SBA for Stages I, II, III and IV is estimated at 50–60%, 40–55%, 10–40% and 3–5%, respectively [8]. By comparison, 5 year OS for CRC is 91, 72 and 14% for localized, regional and distant metastatic disease respectively [33]. This difference in survival is because around a third of patients with SBA present with metastatic disease, most likely due to difficulty in making the initial diagnosis. The workup can be complicated by vague symptoms, tricky endoscopic assessment and limited visibility with radiological techniques [3]. For locoregional disease, the main treatment is surgical resection. R0 resections have been shown to improve prognosis compared to R1 or R2 resections in duodenal tumours, and so adequate lymph node resection should also be performed in jejunal and ileal cancers [34]. There is also an increasing uptake of adjuvant chemotherapy because distant recurrence accounts for 86% of all recurrences [8].

When selecting appropriate patients to undergo CRS–HIPEC, the survival benefit needs to be balanced against the perioperative risks and patient quality of life, especially given the already limited life expectancy observed in the natural course of SBA–PM. Multiple factors need to be considered and the selection process would be best facilitated through a multidisciplinary team discussion that involves the patient to create an individualised treatment plan [35]. Selection factors to consider should include tumour grade, PCI score, time since diagnosis, pre-existing comorbidities and age.

Compared to systemic chemotherapy alone, CRS–HIPEC has the potential to improve the OS for SBA–PM patients. The median OS for SBA–PM patients can be up to 32 months after CRS–HIPEC, compared to up to 19 months with systemic chemotherapy [8] and a mere 2.5 months when managed with supportive therapy [27]. Half of the 12 studies reported a median OS of around 30 months, which is less than the median OS reported for colorectal peritoneal metastases (CRPM) thought to be around 41 months [36]. This is consistent with SBA generally considered as more aggressive and less responsive to systemic chemotherapy [13, 37]. One of the studies in this review had a median OS of just 9 months for SBA–PM, which is on par with OS for palliative chemotherapy alone; however, half of the patients (4/8) in the report experienced Clavien–Dindo Grade III/IV morbidity, which may have contributed to the lower OS rate [23]. The wide range of OS amongst these studies could be related to study heterogeneity. The studies had long inclusion periods spanning from 1989 to 2017 and a wide range of interventions including chemotherapy, adjuvant chemotherapy, HIPEC and EPIC. Just over 50% of patients across the included studies underwent neoadjuvant chemotherapy. This suggests a selection bias as patients who undergo upfront CRS–HIPEC are likely to have a lower PCI compared to those who undergo neoadjuvant chemotherapy. Unfortunately, there was insufficient data to directly compare these two cohorts. One of the included studies did not include any data on systemic chemotherapy treatments [22].

Interestingly, the 5-year survival rate for SBA without PM has been reported to be as low as 36% but was noted to steadily improve throughout the reporting period from 2005 to 2017 [5]. In another study, 5-year survival was understandably shown to be directly related to stage (Stage I – 79%, Stage II – 58%, stage III – 38%) [38]. In this review, the 5-year survival rate ranged between 25 and 40% from a total of 164 patients with SBA–PM who underwent CRS–HIPEC, which is similar to the 5-year survival of 23–52% reported for CRPM [14]. Thus, for adequately treated SBA–PM, the prognosis may not be as poor as once thought. As CRS–HIPEC becomes more widely available for more common malignancies such as CRPM [39, 40], CRS–HIPEC should also be appropriate in SBA–PM patients.

However, whilst it has the potential to improve survival in a highly selected group of patients compared to palliative chemotherapy alone, treatment failure following CRS–HIPEC is common. Five included studies reported median PFS between 7 and 14 months after CRS–HIPEC [18, 21, 23, 25, 28], which is not better than the reported median PFS of 3–11 months following systemic chemotherapy as a single agent treatment [13].

There is an intrinsic risk of morbidity and mortality associated with CRS–HIPEC [41], which needs to be balanced against the expected survival benefits of the procedure. CRS can involve several peritonectomy and multivisceral resections, with a risk of inadvertent injury to surrounding structures. A study of 147 patients who underwent CRS–HIPEC for PM of appendiceal or colorectal origin found that small bowel resection and the number of anastomoses performed was significantly correlated with gastrointestinal morbidity [42]. The addition of high concentration chemotherapy and hyperthermia may impact the physiological healing process and contribute to the incidence of gastrointestinal complications such as anastomotic leaks and enterocutaneous fistulas [43]. Of the 164 patients across the 3 studies included in this systematic review, there were 3 cases of mortality (1.8%) and the overall rate of Grade III/IV Clavien–Dindo morbidity was 21.3%. This is within the same range reported by systematic reviews for CRS–HIPEC for PC of various origins [44]. SBA–PM patients appear to have a similar morbidity rate, reinforcing the importance of patient selection, which must balance fitness for surgery with possible oncologic benefit.

Unsurprisingly, higher rates of morbidity were observed in patients with higher PCI [22], as these patients require more extensive peritonectomy and are more likely to have incomplete cytoreduction. Complications commonly involved the gastrointestinal tract and respiratory system, including peritoneal abscesses, sepsis, intestinal fistula formation and pleural effusions [28]. PCI has been identified as an independent risk factor for gastrointestinal complications after CRS–HIPEC [42] as patients with more peritoneal disease require more extensive surgery. In Mah et al.’s 2019 study of 38 patients with PM from rare aetiologies, of which 4 were small bowel primary malignancies, all who had Grade III/IV complications (34.2%) had a large burden of disease (mean PCI score 29.4) prior to undergoing CRS–HIPEC. Subsequently, patients who have undergone more extensive surgery may require a longer period of recovery in hospital, which is known to be associated with more frequent pulmonary complications [45].

Due to the rarity of SBA–PM, a lot of what is understood about the disease has been extrapolated from CRPM, which is a more common malignancy that has been more thoroughly investigated [Table 5]. The neoadjuvant, adjuvant and intraperitoneal chemotherapeutic agents that have been trialed in SBA–PM patients are similar to those which have a proven benefit in CRPM patients. Currently, based on CRPM evidence, mitomycin C (MMC) and oxaliplatin are the most used HIPEC agents for SBA–PM. However, a 2019 systematic review comparing oxaliplatin vs. MMC in HIPEC for CPRM reported a higher proportion of severe complications following oxaliplatin HIPEC [46]. Additionally, the PRODIGE-7 multicentre randomised trial showed no benefit of oxaliplatin-based CRS–HIPEC vs. CRS alone, albeit using a very short intraperitoneal perfusion time of 30 min [36]. Although there were some limitations with the PRODIGE-7 trial, including the shorter duration of HIPEC, the key component and message is a good cytoreductive surgery with the goal of CC-0 improves outcome. There is also suggestion that MMC should be the agent used for HIPEC, although this needs to be confirmed with a similar design trial as PRODIGE-7. Irrespective, extrapolating data based on CRPM, high quality CRS and HIPEC using MMC would be the agent of choice for SBA–PM patients.

**Table 5:** Comparison of PRODIGE 7 data on CRPM and SBA–PM data from this review.

	Median length of hospital stay, days	Treatment-related deaths
Median age	60	54–58
Male predominance	49%	54–88%
Median PCI	10	10–21.5
Complete macroscopic CRS	89%	62.5–100%
Median OS	41.7 months	9–32 months
1-year OS	86.9%	38–83%
5-year OS	39.4%	25–38%
Median PFS	–	7–14 months
Length of stay	14–27 days	14–16 days
Grade III/IV complications	42%	21.3%

This review is limited by the heterogeneity of the studies, the small sample sizes and the retrospective analysis of data. All of this is likely a reflection of the rare nature of SBA, which has precluded the collection of sufficient data from which treatment options can be evaluated and clinical guidelines can be developed. This highlights the importance of referring SBA–PM patients to a quaternary centre with expertise in CRS–HIPEC for a multidisciplinary discussion to determine a tailored treatment plan.

## Conclusions

SBA–PM is a rare disease with a poor prognosis due to its late detection and poor response to systemic chemotherapy. There is emerging evidence that CRS–HIPEC could be a safe and feasible treatment in select patients with SBA–PM. Best practice will most likely be based upon multicentre retrospective evidence and parallels drawn with CRPM management.

## Appendix 1

### Search strategy

[Small bowel adenocarcinoma AND hyperthermic intraperitoneal chemotherapy] OR [small bowel adenocarcinoma AND HIPEC] OR [small bowel tumour AND hyperthermic intraperitoneal chemotherapy] OR [small bowel tumour AND HIPEC] OR [small bowel adenocarcinoma AND cytoreductive surgery] OR [small bowel tumour AND cytoreductive surgery] OR [small bowel AND HIPEC] OR [small bowel AND cytoreductive surgery] OR [small bowel AND intraperitoneal chemotherapy].
